# Correlation patterns between lifetime mood spectrum, impulsivity, and disordered eating behaviors in candidates to bariatric surgery

**DOI:** 10.1017/S1092852926101060

**Published:** 2026-06-18

**Authors:** Claudia Carmassi, Davide Gravina, Margherita Barbuti, Andrea Bordacchini, Berenice Rimoldi, Debora Andreoli, Andrea Coccoglioniti, Marly Simoncini

**Affiliations:** Department of Clinical and Experimental Medicine, University of Pisa, Italy

**Keywords:** Obesity, bariatric surgery, grazing, night eating, emotional eating, disordered eating behaviors, mood spectrum, mood disorders

## Abstract

**Objective:**

A mutual influence between mood disorders and obesity has been reported. Maladapting eating behaviors associated with mood spectrum swings may play a pivotal role in the development and maintenance of obesity. Considering that, the aim of the present study is to investigate the relationship between problematic eating behaviors, namely emotional eating, grazing and night eating, with impulsivity and lifetime mood spectrum (mood, energy, cognition, rhythmicity and vegetative functions) in severe obese patients seeking bariatric surgery.

**Methods:**

304 obese outpatients were recruited at the Psychiatric Unit of the University Hospital of Pisa. Participants completed psychometric tools including the Emotional Eating Scale (EES), the Italian-Night Eating Questionnaire (I-NEQ), the Grazing Questionnaire (GRAZ), the Barratt Impulsiveness Scale (BIS-11), and the Mood Spectrum Self-Report – lifetime version (MOODS-SR). Descriptive analyses and linear correlation were performed.

**Results:**

The sample was predominantly female (71.4%), with a mean age of 47.5 ± 10.9 years and a mean BMI of 43.3 ± 6.3 kg/m^2^. Impulsivity was positively correlated with depressive and manic mood, energy, and cognitive MOODS-SR domains. Emotional eating, night eating, and grazing were significantly associated with the MOODS-SR total score (r=0.422, p=0.000; r=0.321, p=0.001; r=0.321, p=0.000, respectively) and with specific depressive and manic subdomains of the MOODS-SR.

**Conclusion:**

Loss-of-control eating behaviors were positively associated with lifetime depressive and manic mood spectrum features in bariatric patients, highlighting the importance of assessing these dimensions to promote a tailored and integrated treatment.

## Introduction

Obesity is a chronic complex disease with a multifactorial etiology and severe impact on the quality of life affecting an increasing and relevant proportion of people worldwide.^1^ The overeating that sustains weight gain in obese patients can be determined and maintained by a variety of genetic, environmental, and psychological factors.^2^ Indeed, disordered eating behaviors (DEBs) observed in severely obese patients may be due to the complex interplay of biological factors, such as the central nervous system-mediated reward and driving mechanisms especially for food with high calories and its gratifying properties, as well as of psychological factors such as the social significance of food.^3^ Some of these are the underlying drivers not only for binge-eating but also for other type of DEBs that significantly impact on the disease such as emotional eating, snacking, grazing, and night eating.^4^ In fact, although the clinical characteristics of these problematic eating behaviors are different from each other, they all share the common matrix of a loss of control over food, albeit to different measures and in different ways.^5^ While emotional eating refers to the tendency to overeat in response to negative emotional states,^6^ people with snacking or grazing experience an atypical loss of control over food, manifesting with an intake of small amounts of food in a disorganized and unplanned manner not in response to the hunger cue.^7^ On the other hand, night eating is defined by eating after awakening from sleep or by excessive food consumption after the evening meal with a significant distress or impairment in functioning.^8^ Previous studies have investigated the prevalence of such DEBs in clinical samples of bariatric patients, highlighting their association with a more severe clinical presentations and negative outcomes in bariatric surgery.^9^ ^–^^14^

Similarly, the bidirectional association between obesity and mood disorders such as depression and bipolar disorder is also known, suggesting that each condition may be a risk factor for the other.^15^ ^–^^17^ The interconnection between obesity and mood disorders seems to be mediated by multiple pathophysiological mechanisms, including low-grade systemic inflammation, dysfunction of the hypothalamic–pituitary–adrenal (HPA) axis, and changes in dopaminergic circuits involved in the regulation of craving and reward.^18^ ^–^^22^ Moreover, chronobiological alterations in circadian rhythms, as well as the psychopathological dimensions of emotional dysregulation and impulsivity in bipolar disorder patients, have been suggested to negatively influence certain eating behaviors such as night-eating and emotional eating.^23^ Indeed, some authors hypothesize that obesity may contribute to the neuroprogression of bipolar disorder, influencing its chronic course and therapeutic response.^24^ However, the specific correlation that might exist between the various problematic eating behaviors in patients with severe obesity in relation to the mood spectrum remains not fully clarified. Based on this, the aim of the present study is to investigate the relationship between emotional eating, grazing, and night-eating along with the depressive and manic variants of the mood spectrum in severely obese patients seeking for bariatric surgery.

## Methods

### Sample recruitment

A total sample of 304 obese outpatients was recruited at the University Hospital of Pisa during the preoperative psychiatric clinical evaluation. According to international guidelines,^25^ ^–^^27^ the presurgical assessment of obese patients seeking for bariatric surgery consists of a multidisciplinary evaluation that involves general surgeons, endocrinologists, and psychiatrists. Participants were consecutively evaluated at the Psychiatry Section of the Department of Clinical and Experimental Medicine of the University of Pisa. Participants were excluded if they: (a) were younger than 18 or older than 75 years; (b) had a mood disorder secondary to a medical or neurological condition; (c) were unable to adequately understand the questionnaires because of language barriers or intellectual impairment; or (d) were unable to provide informed consent. The inclusion criteria were represented by all clinical conditions that did not fall within those mentioned above. Sociodemographic information and psychiatric clinical history were thoroughly investigated by a psychiatrist with particular attention to current and lifetime psychiatric diagnosis, and obesity parameters and features such as body mass index (BMI) and weight trend over time. Patients who met the eligibility criteria for the study were asked whether they wanted to participate in the enrolment. Therefore, after explaining the rationale of the study a specific set of self-report scales and questionnaires were administered to the participants to collect data for the purpose of the study. Written informed consent was obtained from all eligible participants after they were provided with a detailed explanation of the study and given the opportunity to ask questions. The study was conducted in accordance with the Declaration of Helsinki and the Ethics Committee of the Azienda Ospedaliero-Universitaria of Pisa approved all recruitment and assessment procedures.

### Assessment instruments

#### Sociodemographic and clinical data

Sociodemographic data and clinical variables (such as current and lifetime psychiatric diagnoses, BMI, current pharmacological treatments) were collected using a Case Report Form specifically designed for this purpose. Furthermore, the Structured Clinical Interview for the Disorders of DSM-5 (SCID-5 CV) was utilized to confirm psychiatric diagnoses of recruited patients according to DSM-5 criteria.^28^

#### Grazing questionnaire

The occurrence of grazing was investigated by using the Italian version of the Grazing Questionnaire (I-GQ).^29^ This psychometric instrument consists of seven items, four of which investigate aspects related to the problematic eating behavior, and three items the feeling of loss of control over food. This questionnaire has shown good psychometric properties^30^ and is used in clinical samples of obese patients.^31^

#### Night eating questionnaire

Night-eating was investigated using the Italian version of the Night Eating Questionnaire (NEQ).^32^ This survey consists of a self-reported instrument composed by 14 items divided into four domains: morning anorexia, evening hyperphagia, mood/sleep, and nocturnal ingestions. The questionnaire is a well-validated instrument commonly used for the screening of night-eating syndrome.^33^

#### Emotional eating scale

Patients were administered the Emotional Eating Scale (EES) to assess the tendency to overfeed in response to emotional states rather than hunger cues.^34^ The survey consists of a 25-item Likert scale and the patient is asked to determine the desire to eat (“no desire at all,” “small desire to eat,” “moderate desire to eat,” “strong desire to eat,” “irresistible desire to eat”) in response to each emotional state (such as depressed, irritable, anxious).

#### Barratt impulsiveness scale

The Barratt Impulsiveness Scale (BIS-11) was used to assess the psychopathological dimension of impulsivity. The BIS-11 consists of 30 items, which participants rate on a 4-point Likert scale: Rarely/Never (=1), Occasionally (=2), Often (=3), and Almost Always/Always (=4). BIS-11 subscales evaluate attentional impulsiveness (inability to focus attention), motor impulsiveness (acting without thinking), and nonplanning impulsiveness (lack of orientation to the future). The scale is a validated self-report tool for assessing impulsivity and has shown good psychometric properties.^35^

#### Mood Spectrum Self-Report—lifetime version

The Mood Spectrum Self-Report—lifetime version (MOOD-SR) was used to investigate lifetime mood spectrum symptomatology. This psychometric questionnaire consists of 161 items that investigate depressive and hypo/manic symptoms in relation to four main subdomains: “mood,” “energy,” “cognition,” “rhythmicity and vegetative functions”.^36^

### Statistical analyses

Descriptive statistics were used to summarize sample characteristics, reported as means and standard deviations (SDs) for continuous variables and as numbers and percentages for categorial variables. The linear correlation between the scale scores was measured with Pearson’s correlation coefficient. All psychometric instruments were analyzed using their total and subscale scores as continuous variables, in line with the exploratory correlational design of the study. Statistical significance was set at *p* < 0.05 (two-tailed). We used the statistical routines of IBM SPSS Statistics for Mac, version 25.0 (SPSS Inc., USA).

## Results

### Socio-demographic characteristics and prevalence of psychiatric disorders of the sample

In the total sample the majority of subjects were females (71.4%) with a mean age of 47.5 ± 10.9 years and a mean BMI of 43.3 ± 6.3 kg/m^2^. Most of the patients were married (68.7%) and employed (79.3%) with a predominance of secondary school (47.4%) or high school diploma (41.1%). In the total sample an overall prevalence of 9.9% was observed for major depressive disorder and a lifetime prevalence of 10.5% for bipolar disorders (3.0% for bipolar disorder type 1, 6.3% for bipolar disorder type 2, 1.3% for cyclothymia). Moreover, the lifetime prevalence of anxiety disorders was found to be 12.8% (9.3% for social anxiety disorder, 7.2% for panic disorder, 3.6% for generalized anxiety disorder). Finally, a prevalence of 8.6% was highlighted for eating disorders (6.3% for binge eating disorder, 2.3% for bulimia nervosa, 1.3% for unspecified eating disorders). The main lifetime psychiatric diagnoses in the total sample are summarized in Table 1.

**Table 1.** Lifetime Psychiatric Diagnoses of the Sample (*N* = 304) Table 1. long description.

Major depressive disorder (*n*, %)	30 (9.9%)
Bipolar disorders (*n*, %) Bipolar 1 disorder Bipolar 2 disorder Cyclothymia	32 (10.5%) 9 (3.0%) 19 (6.3%) 4 (1.3%)
Anxiety disorders (*n*, %) Social anxiety disorder Panic disorder Generalized anxiety disorder Unspecified anxiety disorder	39 (12.8%) 5 (1.6%) 22 (7.2%) 11 (3.6%) 1 (0.3%)
Eating disorders (*n*, %) Binge eating disorder Bulimia Nervosa Unspecified eating disorder	30 (8.6%) 19 (6.3%) 7 (2.3%) 4 (1.3%)

### Mood spectrum correlations with impulsivity and loss of control eating behaviors

Linear correlations have been performed to assess the influence of the lifetime mood spectrum with the impulsivity dimension and with the occurrence of problematic eating behaviors for this clinical sample. A significantly strong positive correlation has been observed for the “mood,” “energy,” and “cognitive” dimensions of both depressive and maniac subdomains as well as for the “rhythmicity” subdomains of the MOODS-SR with the total score of the BIS (Table 2). Moreover, a significant positive correlation has been identified between all these variables of the MOODS-SR with the “attentional,” “motor,” and “non-planning” impulsivity subscales of the BIS, except for the “cognitive” dimension of the maniac subdomain of the mood spectrum and the “nonplanning” impulsivity (*p* = 0.385). Furthermore, a significantly positive correlation was highlighted for the NEQ total score with the “mood” (*p* = 0.000), “energy” (*p* = 0.000), and “cognitive” (*p* = 0.003) dimensions of the depressive subdomains of the MOODS-SR, as well as for the “energy” dimension of the maniac subdomain and for the “rhythmicity” subdomain (*p* = 0.034) of the scale. Additionally, when evaluating the relationship between grazing and mood spectrum, a positive significant correlation has also been observed between the total score of the grazing questionnaire with the “mood” (*p* = 0.000), “energy” (*p* = 0.000), and “cognitive” (*p* = 0.000) dimensions of the depressive subdomains of the MOODS-SR. Conversely, a weaker association emerged with the manic variables of the mood spectrum, as the only statistically significant correlation between the grazing questionnaire and the maniac subdimension of the MOODS-SR was observed for the maniac “mood” dimension but not for the “energy” (*p* = 0.070) and “cognitive” (*p* = 0.466) ones. Finally, a substantial linkage between emotional eating and mood spectrum has been observed. Indeed, a positive and significant correlation was found between the EES total score with all subdomains of the MOODS-SR scale. An overview of outcomes is reported in Table 2.

**Table 2.** Linear Correlation between MOOD Spectrum with Impulsivity, Night Eating, Emotional Eating, and Grazing Table 2. long description.

	Total BIS r* (Sig.2-tails)	BIS attentional r* (Sig.2-tails)	BIS motor r* (Sig.2-tails)	BIS nonplanning r* (Sig.2-tails)	Total EES r* (Sig.2-tails)	Total NEQ r* (Sig.2-tails)	Total GQ r* (Sig.2-tails)
Mood depr.	0.346 (0.000)	0.391 (0.000)	0.266 (0.000)	0.206 (0.001)	0.398 (0.000)	0.362 (0.000)	0.286 (0.000)
Energy depr.	0.430 (0.000)	0.441 (0.000)	0.338 (0.000)	0.276 (0.000)	0.395 (0.000)	0.325 (0.000)	0.312 (0.000)
Cognit. depr.	0.332 (0.000)	0.377 (0.000)	0.246 (0.000)	0.206 (0.001)	0.378 (0.000)	0.276 (0.003)	0.300 (0.000)
Mood man.	0.287 (0.000)	0.318 (0.000)	0.237 (0.000)	0.159 (0.010)	0.309 (0.000)	0.159 (0.088)	0.198 (0.023)
Energy man.	0.303 (0.000)	0.276 (0.000)	0.322 (0.000)	0.152 (0.015)	0.237 (0.000)	0.246 (0.010)	0.163 (0.070)
Cognit. man.	0.203 (0.001)	0.236 (0.000)	0.224 (0.000)	0.057 (0.385)	0.190 (0.002)	0.075 (0.425)	0.064 (0.466)
Rhytmicity	0.304 (0.000)	0.331 (0.000)	0.306 (0.000)	0.131 (0.034)	0.412 (0.000)	0.198 (0.034)	0.178 (0.041)

Abbreviation: r*,Pearson’s coefficient; BIS, Barratt Impulsiveness Scale; EES, Emotional Eating Scale; NEQ, Night Eating Questionnaire; GQ, Grazing Questionnaire.

### Correlation between emotional eating, night eating, grazing, and impulsivity

When investigating the relationship between emotional eating and impulsivity a significantly positive association was highlighted for the EES total score with the BIS-11 total score (*p* = 0.006), together with a significant correlation with two of the subdimensions of the scale, namely the attentional impulsivity (*p* = 0.000) and the motor impulsivity (*p* = 0.047). In addition, when evaluating the relationship between night eating and impulsivity, a significant correlation was also observed for the NEQ total score with the BIS-11 total score (*p* = 0.003) and its attentive (*p* = 0.001) and motor (*p* = 0.016) subdimensions. Similarly, a significant positive correlation was found for the total score of the grazing questionnaire with the BIS-11 total score (*p* = 0.017), and the attentional (*p* = 0.002) and motor (*p* = 0.026) impulsivity. Finally, no correlation was observed between emotional eating, night eating or grazing with the nonplanning subdomains of the scale. These results are summarized in Table 3.

**Table 3.** Linear Correlation between Disordered Eating Behaviors and Impulsivity Table 3. long description.

	Total BIS r* (Sig.2-tails)	BIS attentional r* (Sig.2-tails)	BIS motor r* (Sig.2-tails)	BIS nonplanning r* (Sig.2-tails)	Total MOODS r* (Sig.2-tails)
Total EES	0.177 (0.006)	0.312 (0.000)	0.127 (0.047)	0.045 (0.486)	0.422 (0.000)
Total NEQ	0.287 (0.003)	0.312 (0.001)	0.230 (0.016)	0.157 (0.120)	0.321 (0.001)
Total GQ	0.217 (0.017)	0.277 (0.002)	0.202 (0.026)	0.071 (0.441)	0.321 (0.000)

Abbreviation: r*,Pearson’s coefficient; BIS, Barratt Impulsiveness Scale; MOODS, Mood Spectrum Self-Report Lifetime Questionnaire; EES, Emotional Eating Scale; NEQ, Night Eating Questionnaire; GQ, Grazing Questionnaire.

### Comparison of problematic eating behaviors in patients with and without anxiety disorders

In order to assess the role of anxiety, two subgroups were identified within the total sample based on the presence or absence of a diagnosis of anxiety disorders according to the SCID-5. An independent samples *t*-test was conducted to compare the mean scores on the disordered eating scales between groups. The results showed significantly higher emotional eating (*t* = −2.41, *p* = 0.017) and grazing (*t* = −2.29, *p* = 0.024), in patients with anxiety disorders compared to those without. However, no statistically significant difference emerged between the two groups regarding night-eating (*t* = −0.95, *p* = 0.344). The results are summarized in the Table 4.

**Table 4.** Comparisons of Emotional Eating, Grazing, and Night Eating between Patients with and without Anxiety Disorders Table 4. long description.

	Patients with anxiety disorders	Patients without anxiety disorders	*t*	*p*	Cohen’s d
EES total score, mean (sd)	31.00 (21.22)	21.52 (21.12)	−2.41	0.017	0.45
GQ total score, mean (sd)	12.33 (6.50)	7.30 (5.20)	−2.29	0.024	0.96
NEQ total score, mean (sd)	16.67 (3.33)	14.53 (5.44)	−0.95	0.344	0.40

Abbreviation: *t*-value, difference between groups; *p*-value, significance; EES, Emotional Eating Scale; GQ, Grazing Questionnaire; NEQ, Night Eating Questionnaire.

## Discussion

Although the relationship between mood disorders and eating disorders has been extensively reported in the literature,^37^ ^–^^39^ to date it has not yet been established which clinical psychopathological aspects may be associated with the problematic eating behaviors we observe in obese patients. In this regard, this study aimed at investigating the correlation between maladaptive eating behaviors in severely obese patients and the psychopathological patterns of lifetime mood spectrum including depressive and manic cognitive, energy, and mood spectrum symptoms including atypical and subthreshold features as well as rhythmicity and vegetative functions.^40^ Previous studies by our research group have highlighted the predisposing role of affective symptoms in the development of DEBs as well as their higher prevalence in patients with a primary diagnosis of bipolar disorder in these clinical samples.^16^ Assuming a dimensional perspective of the bipolar spectrum, mood spectrum symptoms may occur through-out life, sometimes in isolation rather than as part of a temporally circumscribed clinical syndrome.^41^ ^,^ ^42^ These patterns of mood and behaviors may also suggest a circadian misalignment in neurobehavioral regulation, highlighting the importance of chronobiological factors in understanding obesity-related eating disturbances. Indeed, mood disorders are not exclusively characterized by qualitative and quantitative alterations in the affective state but are also closely associated with cognitive impairment that not only contribute to the symptoms but also influence the prognosis.^43^ Consistently, patients with mood disorders and overweight/obesity show alterations in selected brain regions associated with cognitive control, emotional regulation, and reward functions even in the absence of full-blown affective symptomatology.^44^ Many patients with mood spectrum disorders may present residual cognitive alterations, particularly in executive functions, working memory, and behavioral inhibition, which could potentially impact eating behaviors as well as lead to difficulties in emotional regulation.^45^ The same influence can also be observed for energy levels, which fluctuate in relation to mood swings, representing one of the core symptoms such as psychomotor slowness and pragmatic inhibition, or, conversely, hyperactivity that is disproportionate to the subject’s abilities or goals. Another cross-cutting element is represented by circadian rhythms, which regulate several neurovegetative and behavioral functions, such as sleep, appetite, and body temperature. These psychopathological aspects are complexly interconnected through shared neurobiological circuits (such as the dorsolateral prefrontal cortex, the amygdala, and the basal ganglia) and neurochemical systems (dopamine, serotonin, norepinephrine, melatonin) that jointly regulate affectivity, cognitive processes, impulse control, and chronobiology, but also play a crucial role in regulating appetite and hunger/satiety signals.^46^ In the sample of the present study, we observed a strong correlation between impulsivity and mood spectrum symptomatology in severely obese patients: mood, cognition, energy levels of lifetime mood spectrum together with rhythmicity subdomains turned out to be significantly associated with all subtypes of impulsivity, namely attentional impulsivity, motor impulsivity, and nonplanning impulsivity. This result confirms the intuitive clinical evidence that impulsivity plays a fundamental role during mood episodes, such as in manic or hypomanic states, where individuals may act without forethought, but also during depressive phases, where poor decision-making may arise from cognitive disinhibition or attempts to escape negative emotions especially in mixed episodes. Indeed, impulsivity is increasingly recognized as a transdiagnostic feature across the mood spectrum and a pivotal contributing factor to mood instability.^47^

Findings from our research also highlighted how the total mood spectrum scores were significantly correlated with the total scores of DEBs, namely emotional eating, night eating, and grazing. In order to explore in detail which mood features accounted for the problematic eating behaviors in severe obese patients, we also performed separated analysis considering each individual subdomain in relation to disordered eating. Mood, cognition, and energy depressive subdomains together with rhythmicity subdomains of the MOODS-SR yielded a positive and significant correlation with emotional eating, night eating, and grazing. These results are in line with a recent review that analyzed 12 studies of overweight/obese clinical sample from the literature and concluded that prolonged stress and depression symptoms were the most frequent discrete pattern associated with emotional eating related with greater food intake and also influencing the kind of food eaten, even if further studies are needed to fill the gap in understanding the underlying mechanisms of the interconnections among emotional eating, overweight/obesity, depression, and dietary patterns.^48^ Evidence shows that depressive spectrum symptomatology led to increased difficulties in distinguishing emotional arousal from hunger. Therefore, palatable food especially when rich in sugar and fat may become a compensatory source of comfort.^49^ Indeed, this kind of patients may experience a low interoceptive awareness that could lead to respond to emotional arousal with frequent snack consumption or graze eating rather than cognitive emotional processing creating a vicious loop particularly regarding internalizing dysfunction, which is characterized by inner-directed emotional distress such as anxiety and depression.^50^ Moreover, a prospective Brazilian study of 60 surgery patients found that baseline depressive symptom severity predicted higher night-eating scores suggesting for a mood-circadian-dependent component in night-eating syndrome within bariatric cohorts.^51^

On the other hand, in our sample, we interestingly found a weaker even not significant correlation between night-eating and mood and cognitive manic subdomains, as well as for grazing with energy and cognitive manic subdomains. This finding suggests that individuals exhibiting symptoms of the manic spectrum of mood, characterized by increased levels of arousal and energy, may suppress appetite through overstimulation of reward pathways, creating a state in which basic physiological needs such as hunger are overshadowed by intensified attention to goal-oriented behaviors, impulsivity, or grandiosity.^52^ However, mood instability could still lead to a greater propensity for emotional eating, especially when their inappropriate beliefs or goals are disappointed, leading to a sense of frustration or distress.^53^ In fact, in our study a positive correlation between emotional eating and mood, cognition, and energy manic subdomains have emerged.

A further relevant aspect emerging from our findings concerns the role of the “rhythmicity and vegetative functions” domain of the mood spectrum. This dimension encompasses alterations in circadian rhythms, including sleep–wake cycle disruptions, appetite regulation, and daily energy fluctuations. In our sample, rhythmicity was significantly associated with emotional eating, night eating, and grazing behaviors, suggesting that circadian dysregulation may represent a pivotal underlying mechanism linking mood spectrum symptomatology to disordered eating patterns. From a clinical perspective, disruptions to biological rhythms can lead to an imbalance between physiological hunger signals and actual food intake, contributing to irregular eating patterns such as nocturnal ingestion or continuous snacking throughout the day. This is particularly relevant for night-eating behaviors, which are intrinsically characterized by a shift in food intake toward the night-time but may also contribute to broader patterns of loss of control overeating. Furthermore, circadian instability may interact with emotional dysregulation and impulsivity, further exacerbating maladaptive eating behaviors.

Finally, when investigating a potential underpinning mechanism about DEBs and mood spectrum for bariatric populations, our research found a positive and significant correlation of impulsivity dimension with emotional eating, night eating, and grazing. More precisely, we observed how these eating behaviors were significantly associated with attentional and motor impulsivity, rather than nonplanning impulsivity. Therefore, attentional deficits and impulse behavior may represent psychopathological features linked to mood instability, but which also sustain to the loss of control eating behaviors. These results from a severely obese sample are in line with a recent large ongoing web-based prospective study on a general population adult volunteer’s cohort started in France in May 2009 that enrolled 51 394 participants and how impulsivity quantitatively moderated the association between emotional eating and BMI.^54^

## Conclusion

Despite limitations such as the cross-sectional study design, the self-reported questionnaire, and additional factors that may limit the generalizability of results such as the higher prevalence of females in the total sample and the small size of some of the clinical subgroups analyzed, our study highlighted a positive correlation between emotional eating, night-eating, and grazing with depressive and manic lifetime mood spectrum in severely obese population seeking for bariatric surgery. Impulsivity might represent a substantial psychopathological aspect that sustain DEBs in this population. These findings underscore the importance to evaluate subthreshold and full-blown mood spectrum symptomatology in clinical setting to promote an accurate evaluation in order to define more appropriate intervention strategies for severe obese patients. In light of that, additional prospective studies are needed to address tailored weight management intervention to improve the presurgical assessment and the post-surgical bariatric course.

## Long descriptions

### Table 1. Long description

The table presents data for four main categories of psychiatric disorders.

* Major depressive disorder: 30 individuals (9.9%).

* Bipolar disorders: Total of 32 individuals (10.5%). This includes Bipolar 1 disorder (9 individuals, 3.0%), Bipolar 2 disorder (19 individuals, 6.3%), and Cyclothymia (4 individuals, 1.3%).

* Anxiety disorders: Total of 39 individuals (12.8%). This includes Social anxiety disorder (5 individuals, 1.6%), Panic disorder (22 individuals, 7.2%), Generalized anxiety disorder (11 individuals, 3.6%), and Unspecified anxiety disorder (1 individual, 0.3%).

* Eating disorders: Total of 30 individuals (8.6%). This includes Binge eating disorder (19 individuals, 6.3%), Bulimia Nervosa (7 individuals, 2.3%), and Unspecified eating disorder (4 individuals, 1.3%).

Navigate back to Table 1..

### Table 2. Long description

The table presents Pearson correlation coefficients r and significance values in 2-tails for seven mood domains across seven clinical scales. The columns represent Total B I S, B I S attentional, B I S motor, B I S nonplanning, Total E E S, Total N E Q, and Total G Q.

* Mood depressive: Correlates most strongly with Total E E S 0.398 and B I S attentional 0.391.

* Energy depressive: Shows the highest overall correlations, particularly with B I S attentional 0.441 and Total B I S 0.430.

* Cognitive depressive: Strongest correlation with Total E E S 0.378 and B I S attentional 0.377.

* Mood manic: Highest correlation with B I S attentional 0.318 and Total E E S 0.309.

* Energy manic: Highest correlation with B I S motor 0.322 and Total B I S 0.303.

* Cognitive manic: Generally lower correlations, with the highest being B I S attentional 0.236. The correlation with B I S nonplanning 0.057, Total N E Q 0.075, and Total G Q 0.064 are not statistically significant.

* Rhythmicity: Strongest correlation with Total E E S 0.412.

All values are positive, indicating that higher scores in mood spectrum domains correlate with higher levels of impulsivity and disordered eating behaviors.

Navigate back to Table 2..

### Table 3. Long description

The table presents Pearson correlation coefficients r and significance values in parentheses across six columns and four rows.

Columns from left to right:

1. Behavioral Scale (Row labels).

2. Total B I S.

3. B I S attentional.

4. B I S motor.

5. B I S nonplanning.

6. Total M O O D S.

Data Rows:

- Total E E S: 0.177 (0.006) for Total B I S; 0.312 (0.000) for B I S attentional; 0.127 (0.047) for B I S motor; 0.045 (0.486) for B I S nonplanning; 0.422 (0.000) for Total M O O D S.

- Total N E Q: 0.287 (0.003) for Total B I S; 0.312 (0.001) for B I S attentional; 0.230 (0.016) for B I S motor; 0.157 (0.120) for B I S nonplanning; 0.321 (0.001) for Total M O O D S.

- Total G Q: 0.217 (0.017) for Total B I S; 0.277 (0.002) for B I S attentional; 0.202 (0.026) for B I S motor; 0.071 (0.441) for B I S nonplanning; 0.321 (0.000) for Total M O O D S.

Abbreviations used: B I S stands for Barratt Impulsiveness Scale; M O O D S stands for Mood Spectrum Self-Report Lifetime Questionnaire; E E S stands for Emotional Eating Scale; N E Q stands for Night Eating Questionnaire; G Q stands for Grazing Questionnaire.

Navigate back to Table 3..

### Table 4. Long description

The table consists of six columns: Variable, Patients with anxiety disorders, Patients without anxiety disorders, t, p, and Cohen’s d.

Row 1: E E S total score, mean sd. Patients with anxiety disorders: 31.00 21.22. Patients without anxiety disorders: 21.52 21.12. t-value: minus 2.41. p-value: 0.017. Cohen’s d: 0.45.

Row 2: G Q total score, mean sd. Patients with anxiety disorders: 12.33 6.50. Patients without anxiety disorders: 7.30 5.20. t-value: minus 2.29. p-value: 0.024. Cohen’s d: 0.96.

Row 3: N E Q total score, mean sd. Patients with anxiety disorders: 16.67 3.33. Patients without anxiety disorders: 14.53 5.44. t-value: minus 0.95. p-value: 0.344. Cohen’s d: 0.40.

Abbreviations include E E S for Emotional Eating Scale, G Q for Grazing Questionnaire, and N E Q for Night Eating Questionnaire.

Navigate back to Table 4..
